# Achieving multi-modal brain disease diagnosis performance using only single-modal images through generative AI

**DOI:** 10.1038/s44172-024-00245-w

**Published:** 2024-07-10

**Authors:** Kaicong Sun, Yuanwang Zhang, Jiameng Liu, Ling Yu, Yan Zhou, Fang Xie, Qihao Guo, Han Zhang, Qian Wang, Dinggang Shen

**Affiliations:** 1 School of Biomedical Engineering & State Key Laboratory of Advanced Medical Materials and Devices, ShanghaiTech University, Shanghai, 201210 China; 2 Health Management Center, Renji Hospital, School of Medicine, Shanghai Jiao Tong University, Shanghai, 200127 China; 3 Department of Radiology, Renji Hospital, School of Medicine, Shanghai Jiao Tong University, Shanghai, 200127 China; 4 Department of Nuclear Medicine & PET Center, Huashan Hospital, Fudan University, Shanghai, 200040 China; 5 National Center for Neurological Disorders, Shanghai, 201112 China; 6 State Key Laboratory of Medical Neurobiology and MOE Frontiers Center for Brain Science, Shanghai Medical College, Fudan University, Shanghai, 200032 China; 7 Department of Gerontology, Shanghai Jiao Tong University Affiliated Sixth People’s Hospital, Shanghai, 200233 China; 8 Shanghai Clinical Research and Trial Center, Shanghai, 200231 China; 9 Shanghai United Imaging Intelligence Co., Ltd., Shanghai, 201807 China

**Keywords:** Magnetic resonance imaging, Health care, Positron-emission tomography

## Abstract

Brain disease diagnosis using multiple imaging modalities has shown superior performance compared to using single modality, yet multi-modal data is not easily available in clinical routine due to cost or radiation risk. Here we propose a synthesis-empowered uncertainty-aware classification framework for brain disease diagnosis. To synthesize disease-relevant features effectively, a two-stage framework is proposed including multi-modal feature representation learning and representation transfer based on hierarchical similarity matching. Besides, the synthesized and acquired modality features are integrated based on evidential learning, which provides diagnosis decision and also diagnosis uncertainty. Our framework is extensively evaluated on five datasets containing 3758 subjects for three brain diseases including Alzheimer’s disease (AD), subcortical vascular mild cognitive impairment (MCI), and O[6]-methylguanine-DNA methyltransferase promoter methylation status for glioblastoma, achieving 0.950 and 0.806 in area under the ROC curve on ADNI dataset for discriminating AD patients from normal controls and progressive MCI from static MCI, respectively. Our framework not only achieves quasi-multimodal performance although using single-modal input, but also provides reliable diagnosis uncertainty.

## Abstract (web summary)

Kaicong Sun and colleagues design a generative uncertainty-aware AI framework to facilitate brain disease diagnosis using single-modal input. Validated on five datasets comprising thousands of subjects, this approach shows promising results close to using multi-modal input across three types of brain disease.

## Introduction

Medical image classification is essential for brain disease diagnosis^1–4^. Modern medical imaging techniques such as magnetic resonance imaging (MRI) are popularly employed for brain disease diagnosis in clinics. Due to different imaging mechanisms, different modalities can contain complementary information and certain modalities may be more appropriate than other modalities for disease diagnosis or treatment.

However, these modalities might be unavailable in practice due to factors such as cost or radiation dose. Since different imaging modalities often have certain intermodality correlation among high-level feature representations due to shared anatomical structures and/or functional activities, synthesis of unavailable modalities from the available one becomes potentially feasible. Integration of these synthesized modalities can involve modality-specific complementary information for enhanced classification performance.

Image synthesis within or across modalities is a typical image translation task. Generative models, such as variational autoencoders (VAE)^5^, generative adversarial networks (GANs)^6^, normalizing flow^7^, and more recent diffusion models^8^, are intensively investigated on natural images in the literature. However, the aim of natural image synthesis is generally to attain high perceptual quality or high image diversity^9^. The lack of clinical reliability may induce concerns for medical applications.

Most medical image classification studies propose to use available imaging modalities only^10–14^. It is non-trivial to incorporate a reliable generative model into the classification task for improved classification performance. There are efforts^15,16^ trying to integrate unavailable modalities into disease diagnosis. Pan et al.^15^ propose a disease-specific model to jointly handle medical image synthesis and disease classification. Although synthesized images can contain disease-relevant features, their contributions to the follow-up AD diagnosis are not efficient and direct. The reasons are mainly twofold. First, voxel-wise synthesis is a severe ill-posed problem. Using fine-grained voxel-wise synthesis to assist less complicated classification task can induce a large complexity burden, and the mismatch of task granularity can lead to inefficient learning. Second, dense voxel-wise synthesis generally requires a large amount of training data, which poses an additional challenge for medical classification task. In study^16^, the authors propose a synthesis-empowered classification framework named DeepGuide, which bypasses dense voxel-wise synthesis by transferring feature representations of under-performing modality to the ones of better-performing modality based on knowledge distillation using multilayer perceptron (MLP)-based guidance model. However, the performance of DeepGuide is limited by the MLP-based guidance model and, in addition, a simple mean square error loss is insufficient to guide disease-relevant feature transfer across modalities.

Although integrating cross-modality synthesis can bring improvement in classification performance, modality synthesis also induce additional risk on classification trustworthiness. In fact, classification trustworthiness can be quantified by classification uncertainty. Existing uncertainty-aware models can be categorized into the Bayesian^17–19^ and non-Bayesian^20–23^ approaches. The Bayesian approaches replace deterministic weights of the network by a posterior distribution of the weights given the training data. During inference, the predictive distribution of the unknown label is calculated by the expectation under the posterior distribution of all the possible configurations of the weights. To reduce the computational cost, Monte Carlo dropout (MC dropout)^24^ is introduced as Bayesian approximation by applying the dropout layer in the model, and uncertainty is generated by running the model multiple times during inference. To avoid estimating the distribution of network weights, non-Bayesian approaches have been proposed in the literature, including but not limited to ensemble-based methods^20,21^, M Heads^25^, and deterministic uncertainty methods^22,23^ (which estimate the uncertainty directly). Recently, Han et al.^23^ propose a multiview classification network based on variational Dirichlet and evidence-level fusion, which achieves accurate and reliable uncertainty estimation.

In this work, we propose a synthesis-empowered uncertainty-aware classification framework for brain disease diagnosis. Our model aims to (1) achieve multi-modal classification performance although using single-modal input based on modality synthesis and (2) provide classification uncertainty based on evidential learning. Instead of performing voxel-wise synthesis, we adopt feature-level imputation, which reduces synthesis complexity and leads to more efficient classification enhancement. Our contributions can be summarized in four-fold. First, our framework is built on a two-stage training scheme for I) disease-relevant multi-modal feature representation learning and II) feature representation transfer. In Stage I, the branches of all the modalities are trained jointly. Our model not only learns disease-related feature representation of each modality, but also aligns features of different modalities based on joint classification. In Stage II, our model synthesizes the features of other modalities from the single input modality using 3D CNN-based encoders, maintaining the rest of the model untouched from Stage I. Second, we propose a hierarchical feature similarity matching scheme applied on multi-level features in Stage II to achieve more efficient and reliable feature transfer. Third, our synthesis-empowered classification framework supports uncertainty estimation based on evidential learning. The uncertainties of all the available and synthesized modalities are integrated based on Dempster-Shafer theory. The estimated uncertainty reveals classification confidence and classification trustworthiness. Lastly, we have comprehensively evaluated our framework on 3758 subjects for three brain diseases, i.e., Alzheimer’s disease, subcortical vascular mild cognitive impairment, and MGMT promoter methylation status in glioblastoma patients from different aspects. Our framework shows promising classification performance close to the case of employing complete multi-modal data and provides accurate classification uncertainty, which can potentially reveal classification correctness.

## Methods

**Fig. 1:**
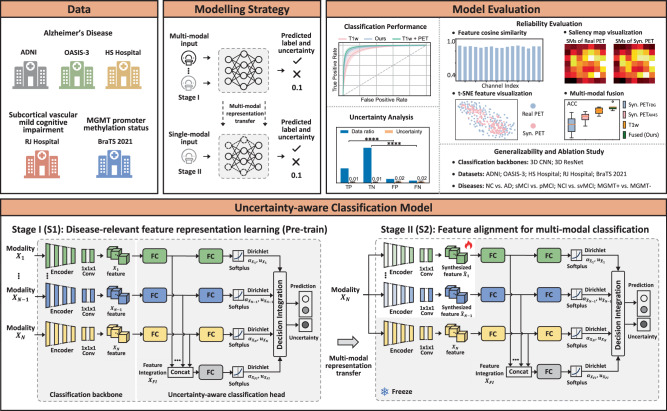
Overview of the study. Multiple cohorts are employed to evaluate our classification framework for multiple brain diseases including Alzheimer’s disease (AD), subcortical vascular mild cognitive impairment, and prediction of O[6]-methylguanine-DNA methyltransferase (MGMT) promoter methylation status. Our framework is evaluated for normal cognition (NC) vs. AD, static mild cognitive impairment (sMCI) *vs*. progressive MCI (pMCI), subcortical vascular mild cognitive impairment (svMCI) *vs*. subcortical vascular disease with no cognitive impairment (NCI), MGMT promoter methylation status (methylated or unmethylated) in aspects of classification performance in terms of area under the ROC curve (AUC), accuracy (ACC), sensitivity (SEN), specificity (SPE), and F1-score (F1) under 5-fold cross-validation, reliability evaluation, uncertainty analysis, ablation study, and generalizability evaluation. The framework is built on a two-stage training scheme: (1) Stage I (S1) aims to learn modality-specific disease-relevant feature representations using real multi-modal data, based on which the classification backbone and classification head of each modality are well established; (2) Stage II (S2) takes single-modal input and performs representation transfer to align the synthesized features with the reference ones from S1. To focus on feature transfer, the branch of available modality (marked in yellow) and the classification heads of all the modalities are borrowed from S1 and frozen in S2 (marked by gray background). To achieve efficient feature alignment, hierarchical similarity matching between the reference features (in S1) and synthesized features (in S2) in the classification backbone and classification head is imposed. More details are given in section “Two-stage training scheme and hierarchical feature similarity matching''.

We provide the overview of our study in Fig. 1. Our framework is evaluated on multi-site multi-modal data for different classification tasks from perspectives of classification performance, synthesis reliability, uncertainty analysis, ablation study, and generalizability evaluation.

### Network architecture

The proposed model architecture consists of two parts, including classification backbone and classification head as demonstrated in Stage I (S1) of Fig. 1. The classification backbone extracts modality-specific disease-relevant features. In this work, we perform an evaluation using two different backbones: (1) A 5-layer 3D CNN encoder with channels of 16-32-64-128-256. In each layer, the kernel size is set as 3 × 3 × 3 followed by an Instance Normalization and a Leaky ReLU. The first layer has a stride size of 1, and the rests have a stride size of 2. A 1 × 1 × 1 layer is applied at the end of the classification backbone to squeeze the channels to 16. Different from S1, in S2 we apply two cascaded Convs in each layer of the unfrozen branches to facilitate synthesis ability; (2) 3D ResNet^26^. We employ ResNet18 for AD diagnosis while ResNet10 for vascular cognitive impairment and MGMT promoter methylation status due to less amount of training data for the latter two diseases. Limited by the GPU memory, we use channel numbers of 32-64-128-256 instead of the original setup of 64-128-256-512. With regard to the classification head, we utilize two fully connected (FC) layers for each modality, and the outputs of the first FC layers of all the modalities are concatenated to construct the feature integration (FI) branch as marked in dark gray in Fig. 1. The output of the second FC layer is passed to the Softplus, instead of the commonly used Softmax, to provide the evidence of individual modality and enable uncertainty estimation. Based on the evidence, belief mass and uncertainty mass of each modality can be directly obtained by means of Dirichlet distribution. The beliefs and uncertainty masses of all the modalities are integrated to obtain an aggregated classification decision and classification uncertainty. More details are given in the section “Uncertainty estimation”.

### Two-stage training scheme and hierarchical feature similarity matching

To obtain reliable disease-relevant feature synthesis, we propose a two-stage training scheme as demonstrated in Fig. 1. In Stage I (S1), all the multi-modal data in the training dataset are utilized to pretrain a model, which learns modality-specific features for disease classification. Note that if a certain modality is missing during training, the corresponding branch will not be updated. In Stage II (S2), our model takes single-modal input, and all the model weights, except the classification backbones of the synthesized modalities (i.e., encoders and the follow-up 1 × 1 × 1 Convs as marked in the white block in Fig. 1), are loaded from the pretrained model in S1 and frozen. To transfer the modality-specific prior from S1 to S2 more effectively, we impose similarity match betweeen S1 and S2 not only on the synthesized features out of the classification backbone, but also on the high-level features in the classification head. In addition, in the first half training epochs of S2, the classification loss is calculated only based on the synthesized modalities, namely the first *N* − 1 branches, to facilitate disease-related feature transfer; in the second half epochs, the available modality, namely the *N*th branch (marked in yellow), and the feature-integration branch (marked in dark gray) are involved for label prediction to further fine-tune the synthesized features.

### Uncertainty estimation

Inspired by the work of ref. ^27,28^, we employ evidential deep learning to quantify classification uncertainty. Evidence in this context is interpreted as the estimate of support from training data in favor of a sample to be classified as a certain label. The principle of evidential-based classification is based on Dempster-Shafer Evidence Theory (DST)^29,30^, which is a generalization of the Bayesian theory to subjective probabilities. In DST, a belief mass is assigned to each possible label, and it allows beliefs from different modalities to be combined to obtain a new belief that considers multi-modal evidence. Later, Subjective Logic (SL)^31^ associates belief distribution with Dirichlet distribution which allows one to use evidence to quantify belief and uncertainty by means of Dirichlet parameter. Specifically, when assigning belief mass *b*_*k*_ (*b*_*k*_ > 0) to *K* class labels in conjunction with the uncertainty mass *u* (*u* > 0), we obtain the *K* simplex formulated as

1 $$u+ {\sum}_{k=1}^{K}{b}_{k}=1,$$

where belief mass *b*_*k*_ = (*α*_*k*_ − 1)/*S* with *α*_*k*_ being the corresponding Dirichlet parameter for the *k*th label which is induced by the evidence *e*_*k*_ = *α*_*k*_ − 1 and $S={\sum }_{k = 1}^{K}{\alpha }_{k}$ being the Dirichlet strength. The above definitions are for single modality. In our concept, we intend to build multi-modal synthesis based on the acquired single-modal data, and integrate the synthesized modalities with the acquired modality for improved classification. Therefore, the proposed classification head will learn the evidence parameters ${{{{{{{\boldsymbol{{e}}}}}}}^{{X}_{n}}}}=\{{e}_{1}^{{X}_{n}},\cdots \,,{e}_{K}^{{X}_{n}}\}$ for all the modalities {*X*_1_, ⋯ , *X*_*N*_} (including synthesized and acquired ones). The beliefs $\{{{{{{{{\boldsymbol{{b}}}}}}}^{{X}_{1}}}},\cdots \,,{{{{{{{\boldsymbol{{b}}}}}}}^{{X}_{N}}}}\}$ and uncertainties $\{{u}^{{X}_{1}},\cdots \,,{u}^{{X}_{N}}\}$ of all the modalities are integrated based on Dempster’s combination rule^23^ to provide the ultimate aggregated belief and uncertainty. Formally, the belief ${{{{{{{{\boldsymbol{b}}}}}}}}}^{{X}_{n}}$ and the corresponding uncertainty ${u}^{{X}_{n}}$ of the *X*_*n*_th modality construct the probability mass ${{{{{{{{\boldsymbol{M}}}}}}}}}^{{X}_{n}}=\{{{{{{{{{\boldsymbol{b}}}}}}}}}^{{X}_{n}},{u}^{{X}_{n}}\}$ with ${{{{{{{{\boldsymbol{b}}}}}}}}}^{{X}_{n}}=\{{b}_{1}^{{X}_{n}},\cdots \,,{b}_{K}^{{X}_{n}}\}$. The combined mass ${{{{{{{\boldsymbol{M}}}}}}}}=\{{\{{b}_{k}\}}_{k = 1}^{K},u\}$ of two independent sets of probability mass {***M***^1^, ***M***^2^} based on the Dempster’s combination rule is formulated as:

2 $${b}_{k}=\frac{1}{1-C}\left({b}_{k}^{1}{b}_{k}^{2}+{b}_{k}^{1}{u}^{2}+{b}_{k}^{2}{u}^{1}\right),\,u=\frac{1}{1-C}{u}^{1}{u}^{2}.$$

According to Eq. (2), the beliefs and uncertainties of different modalities can be integrated. The label with largest belief is considered as diagnosis decision, and the combined uncertainty is taken as final uncertainty.

### Training loss

The loss function for S1 consists of components for all the modalities and also the integrated ones as formulated below:

3 $${L}_{S1}= {\sum}_{{X}_{n}={X}_{1}}^{{X}_{N}}L({{{{{{{{\boldsymbol{\alpha }}}}}}}}}_{{X}_{n}})+L({{{{{{{{\boldsymbol{\alpha }}}}}}}}}_{FI})+L({{{{{{{{\boldsymbol{\alpha }}}}}}}}}_{DI})= {\sum}_{i=1}^{N+2}L({{{{{{{{\boldsymbol{\alpha }}}}}}}}}_{i}),$$

with ${{{{{{{{\boldsymbol{\alpha }}}}}}}}}_{{X}_{n}}$ being the Dirichlet parameter for modality *X*_*n*_. ***α***_*FI*_ and ***α***_*DI*_ represent the Dirichlet parameters for the feature integration branch and the final decision integration block, respectively. Each loss component contains two terms:

4 $$L({{{{{{{{\boldsymbol{\alpha }}}}}}}}}_{i})={L}_{waCE}({{{{{{{{\boldsymbol{\alpha }}}}}}}}}_{i})+KL[D({{{{{{{{\boldsymbol{p}}}}}}}}}_{i}| {\tilde{{{{{{{{\boldsymbol{\alpha }}}}}}}}}}_{i})\parallel D({{{{{{{{\boldsymbol{p}}}}}}}}}_{i}| {{{{{{{\bf{1}}}}}}}})].$$

The first term is the weighted adjusted cross-entropy (waCE), and the second term is the KL divergence between probability distribution $D({{{{{{{{\boldsymbol{p}}}}}}}}}_{i}| {\tilde{{{{{{{{\boldsymbol{\alpha }}}}}}}}}}_{i})$ and *D*(***p***_*i*_∣**1**). ***p***_*i*_ denotes the class assignment probability of the *i*th modality. ${\tilde{{{{{{{{\boldsymbol{\alpha }}}}}}}}}}_{i}$ is the adjusted Dirichlet parameter (definition see below). Our proposed waCE is defined as follows:

5 $${L}_{waCE}({{{{{{{{\boldsymbol{\alpha }}}}}}}}}_{i}) = 	 \int\left[{\sum}_{k=1}^{K}{w}_{k}^{i}{y}_{k}^{i}\log \left({p}_{k}^{i}\right)\right]\frac{1}{B({{{{{{{{\boldsymbol{\alpha }}}}}}}}}_{i})}{\prod}_{k=1}^{K}{\left({p}_{k}^{i}\right)}^{{\alpha }_{k}^{i}-1}\,d{{{{{{{{\boldsymbol{p}}}}}}}}}_{i}\\ = 	 {\sum}_{k=1}^{K}{w}_{k}^{i}{y}_{k}^{i}\left(\psi ({S}_{i})-\psi \left({\alpha }_{k}^{i}\right)\right),$$

where *ψ*( ⋅ ) represents the *digamma* function and *B*( ⋅ ) is the multivariate *beta* function. ${y}_{k}^{i}$ denotes the label of the *k*th class for the *i*th modality, and ${w}_{k}^{i}$ is the corresponding weight which is calculated as the ratio between the number of negative and positive labels. The KL divergence loss is formulated as

6 $$KL[D({{{{{{{{\boldsymbol{p}}}}}}}}}_{i}| {\tilde{{{{{{{{\boldsymbol{\alpha }}}}}}}}}}_{i})\parallel D({{{{{{{{\boldsymbol{p}}}}}}}}}_{i}| {{{{{{{\bf{1}}}}}}}})]= 	 \,log\left(\frac{\Gamma \left({\sum }_{k = 1}^{K}{\tilde{\alpha }}_{ik}\right)}{\Gamma (K){\prod }_{k = 1}^{K}\Gamma ({\tilde{\alpha }}_{ik})}\right)\\ 	 +{\sum}_{k=1}^{K}({\alpha }_{ik}-1)\left[\psi ({\tilde{\alpha }}_{ik})-\psi \left(\mathop{\sum}_{k=1}^{K}{\tilde{\alpha }}_{ik}\right)\right],$$

where Γ( ⋅ ) denotes the *gamma* function, and the adjusted Dirichlet parameter ${\tilde{{{{{{{{\boldsymbol{\alpha }}}}}}}}}}_{i}$ is defined as ${\tilde{{{{{{{{\boldsymbol{\alpha }}}}}}}}}}_{i}={{{{{{{{\boldsymbol{y}}}}}}}}}_{i}+{{{{{{{{\boldsymbol{\alpha }}}}}}}}}_{i}(1-{{{{{{{{\boldsymbol{y}}}}}}}}}_{i})$.

Different from loss *L*_*S*1_, in S2 we apply multi-level similarity matching on the synthesized features in addition. Specifically, *L*_*S*2_ consists of hierarchical constraints including dissimilarity penalty in the classification backbone *L*_*B*_ and classification head *L*_*H*_. Therefore, *L*_*S*2_ is expressed as

7 $${L}_{S2}={L}_{S1}+{\lambda }_{B}{L}_{B}+{\lambda }_{H}{L}_{H},$$

where *λ*_*B*_ and *λ*_*H*_ are tunable hyperparameters. For each level of similarity constrains, we perform point-wise similarity match by mean square error and vector-wise similarity match by cosine similarity. For instance, *L*_*B*_ is formulated as

8 $${L}_{B}={L}_{MSE}+{\lambda }_{CS}{L}_{CS},$$

with *λ*_*CS*_ being a scalar weighting parameter.

### Implementation details

We train the individual models using the datasets reported in Table 1. All the models are built on the same framework with the best hyperparameters chosen according to AUC, SEN, and SPE on the validation data. Specifically, for the ADNI dataset, we employ Adam as the optimizer, and set the training epochs as 30 for each training stage. In S1, the learning rate is set as 1 × 10^−4^ for the first half epochs, and then decayed to 1 × 10^−5^. In S2, we set the learning rate as 1 × 10^−3^ for the first half epochs, and then decrease it to 1 × 10^−4^. The mini-batchsize is set as 10. The weighting parameters *λ*_*B*_ and *λ*_*H*_ are selected as 1, 1 × 10^−2^, and 2 × 10^−4^, respectively. We set *λ*_*CS*_ as 5 in both the *L*_*B*_ and *L*_*H*_. For OASIS-3 and HS Hospital datasets, we initialize the model weights using the pretrained model based on ADNI. The learning rate is set as 5 × 10^−5^, and mini-batch size is set as 4. With respect to applications of svMCI and MGMT promoter methylation status, both mini-batchsizes are set as 4, and learning rate is set as 1 × 10^−4^, and decayed by 0.1 after half of the epochs. All the weighting parameters in loss functions remain the same as for ANDI.

**Table 1:** Study population and characteristics

Dataset	Age (mean ± std)	Gender (Male / Female)	Education (mean ± std)	MMSE (mean ± std)
Alzheimer’s disease				
ADNI				
NC [*n* = 868]	72.4 ± 6.6	381/487	16.5 ± 2.5	29.1 ± 1.1
AD [*n* = 407]	74.9 ± 7.8	229/178	15.3 ± 2.9	23.2 ± 2.2
sMCI [*n* = 575]	72.3 ± 7.6	348/227	16.2 ± 2.7	28.1 ± 1.6
pMCI [*n* = 279]	73.9 ± 7.0	161/118	15.9 ± 2.8	26.8 ± 1.8
MRI_*T*1*w*_ [*n* = 2129]	73.0 ± 7.2	1119/1010	16.1 ± 2.7	27.4 ± 2.7
PET_*FDG*_ [*n* = 1380]	73.3 ± 7.2	772/608	16.1 ± 2.7	27.2 ± 2.8
PET_*AV*45_ [*n* = 1027]	72.3 ± 7.2	525/502	16.4 ± 2.6	27.64 ± 2.7
OASIS-3				
NC [*n* = 300]	68.3 ± 8.7	97^*^/190^*^	15.8 ± 2.5	29.1 ± 1.3
AD [*n* = 257]	76.0 ± 7.8	142^*^/106^*^	14.73 ± 3.1	24.2 ± 4.1
MRI_*T*1*w*_ [*n* = 557]	71.8 ± 9.1	239^*^/296^*^	15.3 ± 2.86	26.9 ± 3.8
PET_*AV*45_ [*n* = 105]	73.3 ± 8.3	37^*^/56^*^	15.8 ± 2.5	27.3 ± 3.5
HS Hospital				
NC [*n* = 140]	64.2 ± 8.5^*^	56/84	12.2 ± 3.1	28.2 ± 1.7
AD [*n* = 91]	64.0 ± 8.0^*^	36/55	9.0 ± 4.1	18.0 ± 5.0
MRI_*T*1*w*_ [*n* = 231]	64.1 ± 8.2^*^	92/139	10.5 ± 4.0	22.7 ± 6.4
PET_*FDG*_ [*n* = 231]	64.1 ± 8.2^*^	92/139	10.5 ± 4.0	22.7 ± 6.4
PET_*AV*45_ [*n* = 231]	64.1 ± 8.2^*^	92/139	10.5 ± 4.0	22.7 ± 6.4
Subcortical vascular mild cognitive impairment				
RJ Hospital				
NCI [*n* = 109]	65.8 ± 7.6	95/14	11.3 ± 3.3	28.7 ± 1.3^*^
svMCI [*n* = 147]	65.0 ± 7.0	109/38	10.0 ± 2.8	27.3 ± 2.0^*^
MRI_*T*1*w*_ [*n* = 256]	65.3 ± 7.3	204/52	10.6 ± 3.1	27.9 ± 1.9^*^
MRI_*FLAIR*_ [*n* = 256]	65.3 ± 7.3	204/52	10.6 ± 3.1	27.9 ± 1.9^*^
MGMT promoter methylation status				
BraTS 2021				
MGMT- [*n* = 278]	–	– / –	–	–
MGMT+ [*n* = 307]	–	– / –	–	–
MRI_*T*1*w*_ [*n* = 585]	–	– / –	–	–
MRI_*T*1*Gd*_ [*n* = 585]	–	– / –	–	–
MRI_*FLAIR*_ [*n* = 585]	–	– / –	–	–
MRI_*T*2*w*_ [*n* = 585]	–	– / –	–	–

Our study includes three medical classification tasks: Alzheimer’s disease, subcortical vascular mild cognitive impairment (svMCI), and MGMT promoter methylation status. We evaluate our framework for AD diagnosis using the public ADNI and OASIS-3, as well as our in-house data from HS Hospital. For svMCI diagnosis and the prediction of MGMT promoter methylation status, we employ in-house data from RJ Hospital and the public BraTS 2021, respectively.

*Incompletely recorded.

### Reporting summary

Further information on research design is available in the Nature Portfolio Reporting Summary linked to this article.

## Results

### Study design and participants

Our framework is evaluated on three brain disease classification tasks, i.e., Alzheimer’s disease (AD), subcortical vascular mild cognitive impairment (svMCI), and prediction of MGMT promoter methylation status in glioblastoma patients. We summarize the study population and characteristics in Table 1. AD and vascular dementia are the most and second most common forms of dementia^32^, respectively. For AD diagnosis, normal cognition (NC) *vs*. AD as well as static mild cognitive impairment (sMCI) *vs*. progressive MCI (pMCI) classifications are carried out. We collect multi-modal data from the Alzheimer’s Disease Neuroimaging Initiative (ADNI) dataset (*n* = 2129)^33–35^, the Open Access Series of Imaging Studies dataset (OASIS-3, *n* = 557)^36^, and a private cohort (*n* = 231) from the local Huashan (HS) Hospital in Shanghai. To alleviate label imbalance in OASIS-3, we adopt all the AD scans (*n* = 257), and randomly select partial NC scans (n = 300). Subcortical vascular cognitive impairment is the most common form of vascular cognitive impairment^37^. In this work, we attempt to distinguish svMCI from subcortical vascular disease with no cognitive impairment (NCI). We utilize an in-house data (*n* = 256), which contains paired T1w and FLAIR images from local Renji (RJ) Hospital in Shanghai. Gliomas are the most common primary central nervous system malignancies, and glioblastoma is the most aggressive subtype of gliomas^38^. MGMT (O[6]-methylguanine-DNA methyltransferase) promoter methylation status is one of the genetic characteristics of glioblastoma, and the determination of MGMT promoter methylation status can influence treatment decision-making^39^. To identify MGMT promoter methylation status (methylated or unmethylated), we use the public BraTS 2021 dataset (*n* = 585)^39–41^ containing multi-parametric MR images (T1w, T1Gd, T2w, T2-FLAIR), acquired with different clinical protocols and different scanners from multiple institutions.

### Data preprocessing and data splitting

We employ publicly available ADNI, OASIS-3, and in-house data from Huashan (HS) Hospital to evaluate our framework for AD diagnosis. T1w images are preprocessed following the standard pipeline, which consists of bias field correction using N4ITK^42^, skull-stripping, and affine registration to the MNI space with 1.5*mm* isotropic spacing by SPM^43^. The spatially normalized T1w images are then cropped to 112 × 128 × 112 to remove the background. PET images are first aligned with the corresponding T1w images by rigid registration. The warped PET images are further transformed to the MNI space by the affine transformation matrix obtained from the corresponding T1w images, so that we obtain the spatially aligned paired T1w and PET images.

We collect 256 subjects from Renji (RJ) Hospital. Each subject contains paired T1w and FLAIR images. We adopt the same preprocessing pipeline to obtain spatially aligned image pairs. To be specific, rigid transform is used to align the T1w image with the corresponding FLAIR image. The T1w images are skull-stripped and registered to the MNI space by affine transformation with a spacing of 1 × 1 × 1 mm^3^. The affine transformation matrix and the transformed mask are then applied to the corresponding FLAIR image. In this way, the skull-stripped T1w and FLAIR images have the spatial correspondence in the MNI space. We crop the image to the size of 176 × 208 × 176. It should be noted that this study is approved by the Research Ethics Committee of HS Hospital and RJ Hospital. Due to the retrospective nature of this study, the informed consent is waived.

We utilize the public BraTS 2021 dataset, which have been preprocessed^39–41^. The data released in Task 1 of BraTS 2021 and the labels released in Task 2 construct our training image-label pair. All of the scans have an isotropic voxelsize of 1 × 1 × 1 mm^3^ and are cropped from the original 240 × 240 × 155 to 192 × 192 × 144 to discard the background region.

In the training phase, we randomly split each dataset into five folds. We train the model using three of the five folds and the rest two folds are employed for validation and test, respectively. Based on 5-fold cross-validation, each fold has been exploited once as test data. The same data splitting is used for both the training stages of S1 and S2 to avoid data leakage.

### Classification performance

To evaluate the classification performance of our framework, we compare with the state-of-the-art classification models, which exploit both the available and synthesized modalities, including PatchGAN^44^, DSNet^15^, and DeepGuide^16^ on ADNI dataset. Particularly, PatchGAN and DSNet perform image synthesis for auxiliary modalities, and then extract disease-relevant features from the synthesized images for follow-up classification. DeepGuide synthesizes features of auxiliary modalities instead of images by feature translation between teacher and student networks. Besides we also compare with the single-modal 3D CNN without auxiliary modalities, denoted as 3D CNN (SI), and the multi-modal variant using complete multi-modal data, denoted as 3D CNN (MI). It is worth noting that our model is built on the backbone of 3D CNN, denoted as Ours_3*DCNN*_. More detailed descriptions of model architecture are given in the Method section. All the models are evaluated by area under the ROC curve (AUC), accuracy (ACC), sensitivity (SEN), specificity (SPE), and F1-score (F1) under 5-fold cross-validation.

We summarize the results for NC *vs*. AD and sMCI *vs*. pMCI classifications on ADNI in Table 2. We can see that compared to the single-modal 3D CNN (SI), Ours_3*DCNN*_ achieves significant improvement, i.e., up to 3.5% in AUC and 6.7% in F1-score for NC *vs*. AD classification, and 6.9% in AUC and 7.4% in F1-score for sMCI vs. pMCI classification. Moreover, our model outperforms the state-of-the-art methods by a large margin as well. Although our framework uses single-modal input, it provides promising classification performance close to multi-modal 3D CNN (MI). The receiver operating characteristic (ROC) curves for NC *vs*. AD and sMCI vs. pMCI classifications are given in Supplementary Fig. 1.

**Table 2:** Classification performance for NC *vs*. AD and sMCI vs. pMCI based on 5-fold cross-validation on ADNI dataset

Methods	AUC	ACC	Sensitivity	Specificity	F1-score
NC *vs*. AD					
3D CNN (SI)	0.915 ± 0.011	0.853 ± 0.023	0.784 ± 0.059	0.885 ± 0.050	0.774 ± 0.018
3D CNN (MI)	0.967 ± 0.006	0.907 ± 0.011	0.879 ± 0.045	0.922 ± 0.036	0.858 ± 0.013
PatchGAN (SI+MmIE)	0.921 ± 0.033	0.863 ± 0.038	0.781 ± 0.050	0.895 ± 0.044	0.778 ± 0.051
DSNet (SI+MmIE)	0.926 ± 0.026	0.871 ± 0.028	0.789 ± 0.030	0.909 ± 0.028	0.796 ± 0.041
DeepGuide (SI+MmFE)	0.924 ± 0.016	0.867 ± 0.023	0.743 ± 0.066	0.925 ± 0.043	0.779 ± 0.036
Ours_3*DCNN*_ (SI+MmFE)	0.950 ± 0.010	0.896 ± 0.011	0.864 ± 0.032	0.912 ± 0.021	0.841 ± 0.023
sMCI *vs*. pMCI					
3D CNN (SI)	0.737 ± 0.039	0.697 ± 0.026	0.611 ± 0.085	0.740 ± 0.066	0.566 ± 0.031
3D CNN (MI)	0.803 ± 0.050	0.770 ± 0.039	0.736 ± 0.080	0.787 ± 0.076	0.675 ± 0.052
PatchGAN (SI+MmIE)	0.774 ± 0.014	0.699 ± 0.040	0.665 ± 0.093	0.713 ± 0.091	0.588 ± 0.027
DSNet (SI+MmIE)	0.791 ± 0.018	0.723 ± 0.010	0.705 ± 0.090	0.732 ± 0.037	0.619 ± 0.049
DeepGuide (SI+MmFE)	0.772 ± 0.038	0.697 ± 0.021	0.677 ± 0.074	0.715 ± 0.053	0.591 ± 0.016
Ours_3*DCNN*_ (SI+MmFE)	0.806 ± 0.041	0.732 ± 0.045	0.734 ± 0.077	0.729 ± 0.065	0.640 ± 0.063

We show the mean value and standard deviation of the 5-fold results. *SI* Single-modal input (T1w); *MI* Multi-modal input (T1w *&* PET_*FDG*_ *&* PET_*AV*45_); *MmIE* Multi-modal image-enhanced (T1w enhanced by synthesized images of PET_*FDG*_ and PET_*AV*45_); *MmFE* Multi-modal feature-enhanced (T1w enhanced by synthesized features of PET_*FDG*_ and PET_*AV*45_).

### Synthesis reliability

Different from PatchGAN^44^ and DSNet^15^ which synthesize images of the unavailable modalities, our framework imputes disease-relevant features of the unavailable modalities. The imputed modality-specific features can be directly forwarded to the classification head without additional feature extraction as required in PatchGAN and DSNet. To validate the reliability of the synthesized features, we have conducted in-depth analysis quantitatively and qualitatively.

**Fig. 2:**
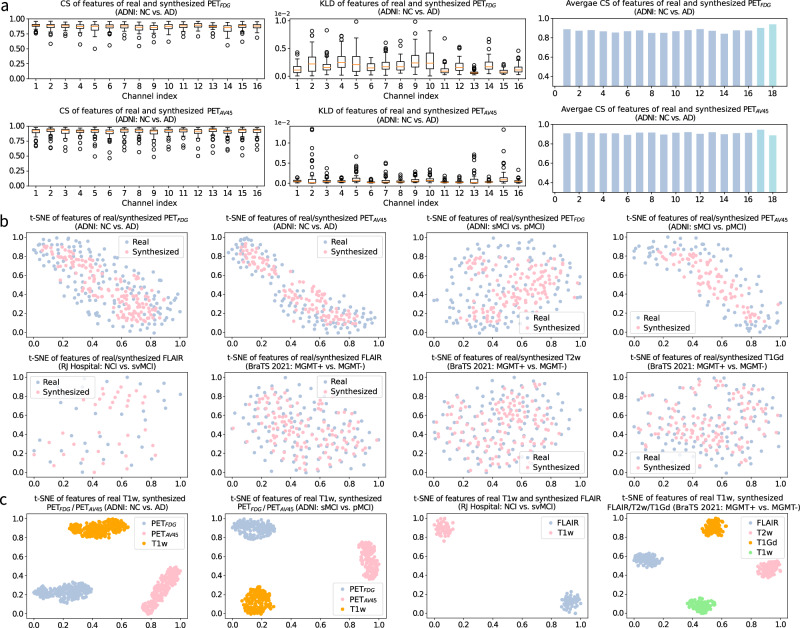
Similarity measure between feature maps of real modalities (obtained in training Stage I), i.e., PET_*FDG*_ and PET_*AV*45_, and the corresponding synthesized ones (obtained in training Stage II) on test data. **a** Boxplots of cosine similarity (CS) and KL divergence (KLD) of the 16-channel backbone features between real and synthesized modalities for normal cognition (NC) *vs*. Alzheimer’s disease (AD) classification across the ADNI test data, with the median values showing in orange bar. The barplots show the average CS of the 16 channels of the classification backbone (average value of each channel as shown in boxplot (**a**) in blue) and additional two bars (in dark green) for the average CS of the features of the two fully connected layers in the classification head. **b** T-SNE visualization of feature representations of real and synthesized modalities for NC *vs*. AD, static mild cognitive impairment (sMCI) *vs*. progressive MCI (pMCI), subcortical vascular disease with no cognitive impairment (NCI) vs. subcortical vascular mild cognitive impairment (svMCI), and MGMT promoter methylation status (methylated or unmethylated) classifications by t-SNE plots. Features are collected from the output of the classification backbone. **c** T-SNE visualization of feature representations of the acquired T1w and synthesized other modalities. It turns out that although synthesized features are generated by T1w, they are complementary to T1w (containing modality-specific patterns).

In Fig. 2, we illustrate quantitative and qualitative similarity measure between features of real (obtained in S1) and synthesized (obtained in S2) modalities. In the boxplots of Fig. 2a, we demonstrate cosine similarity (CS) and Kullback-Leibler Divergence (KLD) measures on the 16 channels of the classification backbone. The CS and KLD between real and synthesized features ***x***_*real*_ and ***x***_*syn*_ are calculated as *CS*(***x***_*syn*_, ***x***_*real*_) = ***x***_*syn*_***x***_*real*_/(∥***x***_*syn*_∥_2_∥***x***_*real*_∥_2_) and *KLD*(***x***_*syn*_, ***x***_*real*_) = ***x***_*real*_(*log****x***_*real*_ − *log****x***_*syn*_), respectively. In the barplots of Fig. 2a, we exhibit the average CS across test subjects for each of the 16 channels (in blue) in the backbone, as well as the two follow-up fully connected layers in the classification head in additional bars (in dark green). It is shown that the average CS reaches up to 0.9 for all the channels in the backbone.

Besides, we also visualize feature representations of real and synthesized modalities using t-SNE on different datasets. Specifically, Fig. 2b illustrates feature representations of the real modalities (by blue dots) and the corresponding synthesized ones (by pink dots). We can see that the real and synthesized features overlap with each other. The quantitative and qualitative evaluations show that the synthesized features are effectively aligned with the real ones, such that the imputed features are plausible and reliable. Furthermore, in Fig. 2c, we exhibit feature representations of different imaging modalities. We can observe that, although imputed from the acquired T1w images, the synthesized features contain modality-specific patterns which are complementary to T1w, so that the integration of synthesized and real features can literally improve classification accuracy and robustness. More similarity evaluation is demonstrated in Supplementary Fig. 2 and Supplementary Fig. 3.

**Fig. 3:**
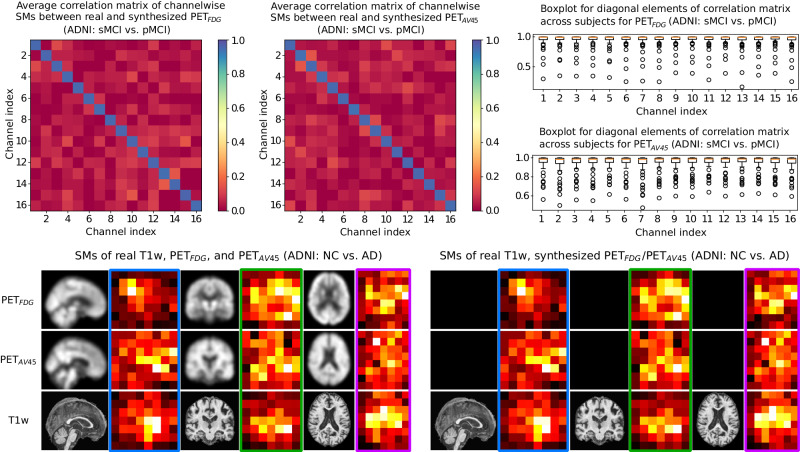
Similarity measure between saliency maps (SMs) of the real and synthesized features on ADNI dataset. Top row left panel: Average correlation matrix of SMs between real and synthesized modalities for static mild cognitive impairment (sMCI) vs. progressive MCI (pMCI) classification. We flatten the SM of each channel (in total 16 channels for backbone feature) and calculate the Pearson correlation coefficients between the real and synthesized modalities for the 16 SMs of each subject. We average the Pearson correlation matrices over the test subjects for PET_*FDG*_ and PET_*AV*45_. The diagonal elements represent the correlation between the real and synthesized SMs of paired channel and all the diagonal elements are close to 1, while non-diagonal elements denote correlation between SMs of unpaired channels and are close to 0. Besides showing the mean value, in the right panel of the top row, we also illustrate statistical analysis of the diagonal elements of the correlation matrices across test data in boxplot, with the median values showing in orange bar. Bottom row: Visualization of SMs of real and synthesized features for a typical case from ADNI. The left panel illustrates SMs obtained using real multi-modal features (three views in training Stage I), while the right panel shows those obtained via synthesized ones (in training Stage II). The unavailable modalities are masked in black, and paired views are marked in the same color.

In addition to similarity analysis on feature maps, we also demonstrate similarity measure on the saliency maps (SMs) of the real and synthesized 16-channel features of the classification backbone in Fig. 3. The SMs are calculated using gradient-based method^45^ over all the test subjects. In the upper part, we demonstrate the average correlation matrix between SMs of real and synthesized features over test subjects. The diagonal elements denote the correlation coefficients between SMs of real and synthesized channel pairs, which is close to 1 and obviously larger than the correlation between unpaired channels (non-diagonal elements). In the right panel, we demonstrate statistical analysis of the diagonal elements of the correlation matrices in boxplots. It is shown that most diagonal elements are above 0.95. In addition to quantitative measure, in the bottom part we visualize the SMs of one subject. Specifically, the left panel illustrates the SMs of real multi-modal features, while the right panel shows the corresponding SMs of the imputed features. It is worthy to note that we demonstrate the average SM across channels for each modality from different views. The unavailable PET_*FDG*_ and PET_*AV*45_ images are masked out in black. We can see that the average SMs of synthesized features are very similar to those of the real modalities. Subtle differences in SMs mainly locate in the regions with less attention, such as at the borders of SMs. Quantitative and qualitative similarity measure on SMs further validates the reliability of our framework. More results can be found in Supplementary Fig. 4 and Supplementary Fig. 5.

**Fig. 4:**
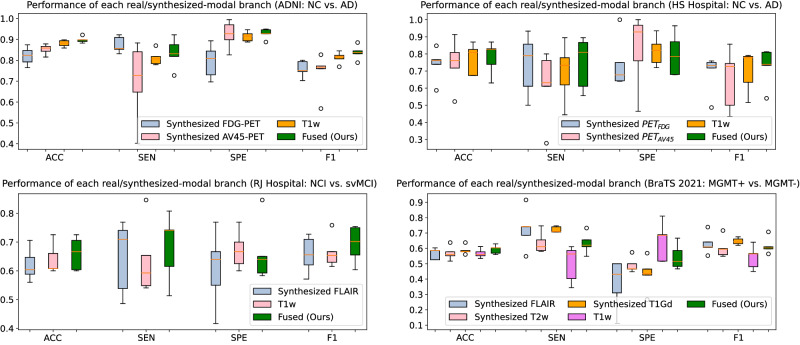
Evaluation of evidence-based multi-modal fusion. We demonstrate the classification performance of each real and synthesized modality (each branch in our model) quantitatively for normal cognition (NC) vs. Alzheimer’s disease (AD), subcortical vascular disease with no cognitive impairment (NCI) *vs*. subcortical vascular mild cognitive impairment (svMCI), and MGMT promoter methylation status (methylated or unmethylated) in terms of accuracy (ACC), sensitivity (SEN), specificity (SPE), and F1-score under 5-fold cross-validation on different datasets. Different modalities are marked in different colors. The orange bars in the boxplots represent the median values for the individual modality.

Moreover, to evaluate the effectiveness of multi-modal fusion, we demonstrate the classification performance of each branch in our model corresponding to the individual real or synthesized modality in Fig. 4. We show ACC, SEN, SPE, and F1-score under 5-fold cross-validation in boxplots for different modalities on multiple datasets. We can see that generally our framework achieves superior performance than the single-modal branch, especially in terms of ACC, by resorting to the multi-modal fusion paradigm, indicating that the synthesized complementary features can be effectively integrated and contribute towards multi-modal performance. Interestingly, some synthesized modality, such as FDG-PET or T1Gd can outperform the available T1w images, and may even achieve slightly better performance than our fused model in terms of sensitivity or specificity. The reason might be due to label imbalance in the training data although we have used weighted adjusted cross-entropy to alleviate this effect. Other techniques, such as oversampling for the minority class could be adopted in the future to further mitigate this effect.

### Uncertainty analysis

**Fig. 5:**
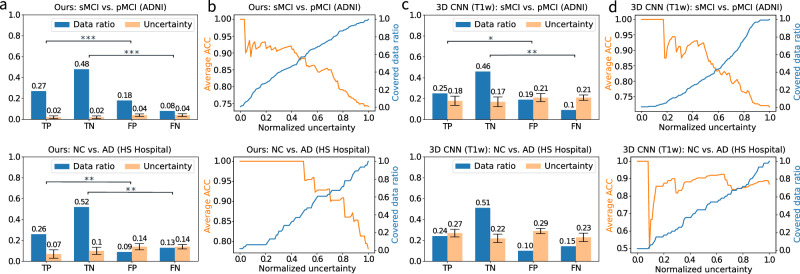
Classification uncertainty analysis. **a**, **b** Uncertainty analysis for our synthesis-empowered classification framework for normal cognition (NC) vs. Alzheimer’s disease (AD) and static mild cognitive impairment (sMCI) *vs*. progressive MCI (pMCI); (c)-(d): Uncertainty analysis for the commonly used single-modal variant. **a** and **c** Plots of confusion matrix with the corresponding average uncertainty on test data. The estimated uncertainty of all the test subjects are categorized according to the confusion matrix, i.e., true positive (TP), true negative (TN), false positive (FP), and false negative (FN). The average uncertainty of each category is shown along with the ratio of covered data. **b** and **d** Plots of average accuracy (ACC) curve and covered data ratio over normalized uncertainty. We compute the average ACC of the subjects, which have the uncertainty above the given threshold. We also show the covered data ratio above this given uncertainty threshold. We denote the two-sided *p*-value *p* < 0.05 as ^*^p < 0.01 as ^**^p < 0.001 as and ^***^p < 0.0001 as ^****^.

One merit of our framework is the classification uncertainty estimation based on integrated modalities. In Fig. 5, we illustrate the uncertainty analysis on ADNI and HS Hospital datasets for AD diagnosis. We demonstrate the uncertainty evaluation for our framework and the single-modal variant in Fig. 5a–d, respectively. In Fig. 5a, we present the uncertainty and covered data ratio with respect to the confusion matrix over test data. Specifically, we calculate the mean and standard deviation of the subject uncertainties for each element of the confusion matrix. We can see that the true positive and true negative predictions have significant lower uncertainty than the corresponding false ones. It suggests that accurate uncertainty estimation can be employed to reveal classification correctness. In Fig. 5b, we demonstrate the average ACC and covered data ratio over normalized uncertainty. The covered data ratio under a given uncertainty threshold is calculated by dividing the number of subjects with uncertainty less than the given threshold by the overall number of subjects. The average ACC is calculated within the subjects whose uncertainties are lower than the threshold. We can observe that, as the uncertainty increases, the average ACC declines, showing that uncertainty can be adopted as a threshold to filter out false predictions and hence obtain improved overall classification performance. Besides, when comparing Fig. 5a with Fig. 5c, our synthesis-empowered classification framework obtains more significant uncertainty difference between the true and false predictions as evaluated by the two-sided *p*-value. This indicates that our framework provides more reliable and robust uncertainty estimation than the single-modal variant. Moreover, our model achieves much lower uncertainty than the single-modal variant, showing that our framework provides not only more accurate diagnosis decision, but also higher diagnosis confidence. More evaluations are available in Supplementary Fig. 6.

### Ablation study

To analyze the effectiveness of the proposed two-stage training scheme and multi-level feature similarity matching, we conduct ablation study on the ADNI data based on 5-fold cross-validation using the same data splitting as mentioned in section “Data preprocessing and data splitting” and summarize the results in Table 3. In fact, Stage I (S1) performs multi-modal classification, aiming for learning modality-specific disease-relevant feature representation, and S2 focuses on feature representation transfer by aligning the synthesized features with the reference ones obtained in S1. It turns out that performing classification and synthesis simultaneously instead of separate stages (denoted as w/o S1) leads to a significant performance drop of 6.7% in average AUC and 12.9% in F1-score. Furthermore, we evaluate the proposed hierarchical constraints on multi-level features in the classification backbone *L*_*B*_ and classification head *L*_*H*_. We can observe that the employment of hierarchical constraints improves the average AUC by 2.5% and the average F1-score by 4.1%.

**Table 3:** Ablation study of our framework for the proposed two-stage training scheme and multi-level feature similarity matching based on the ADNI dataset

Methods	AUC	ACC	Sensitivity	Specificity	F1-score
Ours w/o S1	0.883 ± 0.015	0.803 ± 0.027	0.765 ± 0.055	0.820 ± 0.051	0.712 ± 0.037
Ours w/o *L*_*B*_ & *L*_*H*_	0.925 ± 0.014	0.870 ± 0.021	0.812 ± 0.041	0.897 ± 0.034	0.800 ± 0.038
Ours w/o *L*_*H*_	0.941 ± 0.016	0.885 ± 0.020	0.853 ± 0.044	0.901 ± 0.037	0.825 ± 0.035
Ours	0.950 ± 0.010	0.896 ± 0.011	0.864 ± 0.032	0.912 ± 0.021	0.841 ± 0.023

We show the mean value and standard deviation of the 5-fold results.

### Generalizability evaluation

In the above experiments, we have shown in-depth analysis of the proposed framework in terms of reliability evaluation, uncertainty analysis, and ablation study. Herein, we evaluate the generalizability of our framework for different brain diseases on multiple datasets using different backbones. Particularly, we conduct classification for NC *vs*. AD using public OASIS-3 and private data from HS Hospital, for NCI *vs*. svMCI using private data from RJ Hospital, and for MGMT+ *vs*. MGMT- on public BraTS 2021. We summarize the results in Table 4. For all the tasks, we employ T1w images as the available modality to impute the features of other modalities. To be specific, for NC *vs*. AD, we employ pretrained model based on ADNI as model initialization and fine-tune it on OASIS-3 and HS Hospital data. For a fair comparison, we perform the same model warm-up for single-modal (SI) and multi-modal (MI) variants. It shows that our framework consistently achieves close performance as the multi-modal one although using single-modal input. For NCI *vs*. svMCI and MGMT+ *vs*. MGMT- classifications, our framework obtains similar superiority. The ROC curves are demonstrated in Supplementary Fig. 7.

**Table 4:** Performance of our framework for NC vs. AD, subcortical vascular disease with no cognitive impairment (NCI) *vs*. subcortical vascular mild cognitive impairment (svMCI), and methylated MGMT (MGMT+) vs. unmethylated MGMT (MGMT-) classifications

Methods	AUC	ACC	Sensitivity	Specificity	F1-score
NC *vs*. AD on OASIS-3 (pretrained on ADNI)					
3D CNN (SI)	0.852 ± 0.020	0.772 ± 0.014	0.710 ± 0.091	0.826 ± 0.103	0.743 ± 0.014
3D CNN (MI)	0.885 ± 0.003	0.821 ± 0.007	0.719 ± 0.023	0.912 ± 0.013	0.790 ± 0.011
Ours_3*DCNN*_ (SI+MmFE)	0.890 ± 0.005	0.831 ± 0.004	0.752 ± 0.024	0.900 ± 0.018	0.806 ± 0.008
NC vs. AD on HS Hospital (pretrained on ADNI)					
3D CNN (SI)	0.751 ± 0.081	0.718 ± 0.038	0.639 ± 0.067	0.774 ± 0.043	0.639 ± 0.055
3D CNN (MI)	0.863 ± 0.076	0.839 ± 0.043	0.754 ± 0.086	0.912 ± 0.043	0.778 ± 0.046
Ours_3*DCNN*_ (SI+MmFE)	0.849 ± 0.098	0.783 ± 0.078	0.734 ± 0.085	0.810 ± 0.106	0.730 ± 0.080
NCI vs. svMCI on RJ Hospital					
3D CNN (SI)	0.677 ± 0.036	0.649 ± 0.034	0.634 ± 0.079	0.676 ± 0.099	0.670 ± 0.057
3D CNN (MI)	0.713 ± 0.041	0.676 ± 0.064	0.697 ± 0.066	0.680 ± 0.149	0.708 ± 0.066
Ours_3*DCNN*_ (SI+MmFE)	0.705 ± 0.040	0.660 ± 0.051	0.684 ± 0.105	0.662 ± 0.095	0.693 ± 0.057
MGMT+ vs. MGMT- on BraTS 2021					
3D CNN (SI)	0.574 ± 0.036	0.564 ± 0.022	0.539 ± 0.035	0.591 ± 0.033	0.562 ± 0.025
3D CNN (MI)	0.594 ± 0.030	0.581 ± 0.034	0.602 ± 0.057	0.556 ± 0.029	0.598 ± 0.052
Ours_3*DCNN*_ (SI+MmFE)	0.600 ± 0.023	0.593 ± 0.025	0.634 ± 0.060	0.544 ± 0.074	0.616 ± 0.048

We show the mean value and standard deviation of the 5-fold results. *SI* Single-modal input, *MI* Multi-modal input, *MmFE* Multi-modal feature enhanced.

We further evaluate the generalizability of our framework by using another classification backbone of 3D ResNet^26^ to show that our synthesis-empowered classification framework is not limited to a certain network structure, but is suitable for general network structures. We summarize the results based on 3D ResNet in Table 5. We can see that, our framework consistently outperforms the single-modal variant and approaches the performance using complete modalities for all the classification tasks. This indicates that our framework possesses great generalizability to different diagnosis tasks and different classification backbones.

**Table 5:** Performance of our framework built on the classification backbone of 3D ResNet^26^ for different classification tasks

Methods	AUC	ACC	Sensitivity	Specificity	F1-score
NC *vs*. AD on ADNI					
3D ResNet (SI)	0.905 ± 0.021	0.851 ± 0.020	0.733 ± 0.067	0.904 ± 0.043	0.752 ± 0.030
3D ResNet (MI)	0.956 ± 0.023	0.910 ± 0.036	0.877 ± 0.052	0.925 ± 0.045	0.864 ± 0.044
Ours_3*DResNet*_ (SI+MmFE)	0.949 ± 0.014	0.895 ± 0.022	0.859 ± 0.026	0.913 ± 0.040	0.838 ± 0.026
sMCI *vs*. pMCI on ADNI					
3D ResNet (SI)	0.753 ± 0.035	0.678 ± 0.028	0.628 ± 0.073	0.728 ± 0.069	0.563 ± 0.052
3D ResNet (MI)	0.804 ± 0.051	0.761 ± 0.050	0.747 ± 0.070	0.767 ± 0.080	0.673 ± 0.063
Ours_3*DResNet*_ (SI+MmFE)	0.802 ± 0.041	0.737 ± 0.043	0.734 ± 0.063	0.742 ± 0.072	0.649 ± 0.056
NCI vs. svMCI on RJ Hospitial					
3D ResNet (SI)	0.664 ± 0.049	0.647 ± 0.026	0.683 ± 0.062	0.593 ± 0.086	0.668 ± 0.057
3D ResNet (MI)	0.715 ± 0.030	0.678 ± 0.031	0.741 ± 0.075	0.667 ± 0.083	0.711 ± 0.058
Ours_3*DResNet*_ (SI+MmFE)	0.710 ± 0.037	0.686 ± 0.032	0.731 ± 0.074	0.640 ± 0.081	0.704 ± 0.058
MGMT+ *vs*. MGMT- on BraTS 2021					
3D ResNet (SI)	0.569 ± 0.016	0.559 ± 0.032	0.556 ± 0.096	0.563 ± 0.080	0.563 ± 0.058
3D ResNet (MI)	0.612 ± 0.021	0.589 ± 0.036	0.611 ± 0.055	0.569 ± 0.051	0.607 ± 0.054
Ours_3*DResNet*_ (SI+MmFE)	0.607 ± 0.017	0.601 ± 0.035	0.603 ± 0.065	0.598 ± 0.054	0.602 ± 0.045

We show the mean value and standard deviation of the 5-fold results. *SI* Single-modal input, *MI* Multi-modal input, *MmFE* Multi-modal feature enhanced.

## Discussion

In this work, we propose an uncertainty-aware classification framework, which achieves quasi-multimodal performance using only single-modal images. Compared to most of the classification models which employ only the available modalities, our framework takes advantage of the generative ability of deep learning and reveals the benefits of synthesis-empowered classification for medical disease diagnosis. To achieve the reliable and effective synthesis of complementary/more advanced modalities, our framework synthesizes disease-relevant features of the auxiliary modalities instead of the images based on a proposed two-stage training scheme. Stage I aims to learn disease-relevant feature representations of auxiliary modalities using real multi-modal data. Stage II performs representation transfer between the real and synthesized features under multi-level feature similarity constraints. In such a way, our framework reduces synthesis complexity dramatically and achieves promising classification performance close to the case of employing complete multi-modal data. Moreover, along with predicted label, a reliable classification uncertainty based on multimodal evidential learning is provided, which can potentially reveal classification correctness.

Our framework is evaluated on three brain diseases, including two most common forms of brain cognitive impairment, i.e., Alzheimer’s disease and vascular cognitive impairment, as well as the prediction of MGMT promoter methylation status for glioblastoma. Experimental results show that our framework outperforms the commonly used single-modal variant by 3.5% in AUC for NC *vs*. AD classification, and 6.9% for sMCI *vs*. pMCI classification on ADNI data. Similar phenomenon is observed on OASIS-3 and private HS Hospital data by increasing AUC from 0.852 to 0.890 and from 0.751 to 0.849, respectively, and also on in-house RJ Hospital data from 0.677 to 0.705 for NCI *vs*. svMCI classification, and on BraTS 2021 from 0.564 to 0.600 for MGMT+ *vs*. MGMT- classification.

It is interesting to note that DeepGuide performs slightly better than our method in terms of average specificity for NC *vs*. AD on ADNI data, but its standard deviation in specificity is doubled, and also its sensitivity is much worse than our method. This indicates that DeepGuide seems to be more sensitive to data imbalance. The main reason might lie in the difference of training scheme used in DeepGuide compared to our method. DeepGuide has three training stages and in the last training stage, it trains the classification head while freezes the feature extraction and feature transition parts, which enforces solely the classification head to fit the imbalanced label and might lead to more sensitive classifier. Besides, it also makes the classification highly dependent on the performance of the feature transition, which is performed in the second training stage and is the most difficult and challenging part for synthesis-empowered classification, since the feature transition part is frozen in the final training stage. Another interesting point is the potential impact of incorrect registration of paired modalities in the data preprocessing stage on our framework. Large misregistration between paired modalities can cause difficulty in feature synthesis (Stage II), since feature synthesis is both location- and orientation-sensitive. Degraded synthesis will lead to a performance drop in disease diagnosis. However, for slight misregistration, the impact could be potentially mitigated by the downsampling effect in the encoder. It could be considered as data augmentation, which could improve the robustness and lead to negligible impact on classification performance.

The reliability of feature-level synthesis is extensively evaluated quantitatively and qualitatively by similarity measure on the synthesized features as well as the corresponding saliency maps. Our framework obtains average cosine similarity over 0.9 for the synthesized features, and average Pearson correlation over 0.95 for the saliency maps. Visual assessment based on t-SNE plots and saliency maps coincides with the quantitative evaluation, indicating the effectiveness of our two-stage training scheme and great generative ability of deep learning.

Besides, our framework possesses a merit of reliable uncertainty estimation. We show that uncertainty can be utilized to reveal classification correctness. In fact, accurate uncertainty estimation enables trustworthy AI-guided disease diagnosis, which can assist radiologists to pay attention only to high-uncertainty cases.

Furthermore, our framework is evaluated on multiple datasets using two classification backbones, including the widely used plain 3D CNN and the 3D ResNet. Based on our experiments, both backbones demonstrate consistent superiority of our synthesis-empowered classification framework over other single-modal variants, and achieve similar performance for multiple diseases. Since 3D ResNet has more network parameters and requires more GPU resources, we would recommend to use the 5-layer 3D CNN as the classification backbone in the current scale of training data.

Our framework has several limitations. First, the performance enhancement arising from modality synthesis highly depends on the synthesis ability of the network. In Fig. 2b, we can see that the synthesized features may not behave as diverse as the real features, which can cause performance degradation compared to the multi-modal network. Since currently we use multi-layer 3D CNN to perform synthesis across modalities in Stage II, more advanced synthesis methods such as diffusion models could be adopted to potentially reduce the gap between multi-modal and synthesis-empowered single-modal classifiers. Second, the current model is disease-specific. We assume that different related brain diseases, such as Alzheimer’s disease and vascular cognitive impairment, may contain certain common features. A unified diagnosis model for all the related diseases could exploit the common latent space and benefit from large amount of training data of different diseases. This disease-unified model not only can perform disease diagnosis given a brain image (such as for fast screening), but also can potentially improve diagnosis performance for each disease based on common feature alignment. A unified diagnosis model that is able to handle multiple related brain diseases could be our future work. Third, the current estimated uncertainties for the same disease have different dynamic ranges across cohorts, for example, the classification uncertainties for NC *vs*. AD between ADNI and OASIS-3 datasets as shown in the supplementary Fig. 6a. If the same patient undergoes AD diagnosis in different hospitals, it would be difficult to compare these diagnosis confidence due to different dynamic ranges of estimated uncertainties. Uncertainty-aware classification framework with a dedicated design for a unified uncertainty range across large-scale of multi-center datasets would be of great interest. In the current experiments, we have not unified the spacing and dimension across all the datasets, which could be one limitation of our work. In fact, we did consider to use the same spacing and dimension for all the diseases. However, the released public BraTS data has already been preprocessed and we prefer to keep it as it is. Another reason is that we employ different models for different diseases, and hence the inconsistent spacing and dimension actually have no impact on their individual performance. We chose to crop the background of each dataset to the maximum extent to reduce the required GPU resource for our deep multi-branch 3D network. However, it would be more general to have the same dimension and spacing for all the datasets even for different diseases.

To summarize, we propose an uncertainty-aware classification framework enhanced by disease-relevant feature synthesis of auxiliary modalities. The proposed framework is validated on five datasets including 3758 subjects for three common brain diseases. We show our framework obtains classification performance close to the case of using complete multi-modal data by making use of the generative ability of deep learning, and meanwhile provides reliable classification uncertainty based on multi-modal evidential learning. Our framework contributes to synthesis-empowered trustworthy classification for AI-guided disease diagnosis and shows great potential to be deployed in clinics for different application scenarios.

### Supplementary information

Supplementary Information

Reporting Summary

## Data Availability

This report and its Supplemental Information provide the primary results that underpin the findings of this study. The trained best checkpoints and partial data are available at^46^.

## Code availability

The code of this paper is publicly available at ref. ^47^. It should be used for academic research only.
