# Supplementary material for: Achieving multi-modal brain disease diagnosis performance using only single-modal images through generative AI
Source: Commun Eng. 2024 Jul 10;3:96. doi: 10.1038/s44172-024-00245-w (PMC11236988; doi:10.1038/s44172-024-00245-w)
Supplement: Supplementary file 1 — Supplementary Information [file 44172_2024_245_MOESM1_ESM.pdf]

Supplementary Information: Achieving Multi-modal Brain Disease Diagnosis Performance Using Only Single-modal Images Through Generative AI

Kaicong Sun[1†], Yuanwang Zhang[1], Jiameng Liu[1], Ling Yu[2], Yan Zhou[3], Fang Xie[4, 5, 6], Qihao Guo[7], Han Zhang[1], Qian Wang[1,9], and Dinggang Shen[1,8,9]

[1]School of Biomedical Engineering & State Key Laboratory of Advanced Medical Materials and Devices, ShanghaiTech University, Shanghai 201210, China
[2]Health Management Center, Renji Hospital, School of Medicine, Shanghai Jiao Tong University, Shanghai 200127, China
[3]Department of Radiology, Renji Hospital, School of Medicine, Shanghai Jiao Tong University, Shanghai 200127, China
[4]Department of Nuclear Medicine & PET Center, Huashan Hospital, Fudan University, Shanghai 200040, China
[5]National Center for Neurological Disorders, Shanghai 201112, China
[6]State Key Laboratory of Medical Neurobiology and MOE Frontiers Center for Brain Science, Shanghai Medical College, Fudan University, Shanghai 200032, China
[7]Department of Gerontology, Shanghai Jiao Tong University Affiliated Sixth People's Hospital, Shanghai 200233, China
[8]Shanghai United Imaging Intelligence Co., Ltd., Shanghai 201807, China
[9]Shanghai Clinical Research and Trial Center, Shanghai 200231, China
[†]Corresponding author: Kaicong Sun (sunkc@shanghaitech.edu.cn)

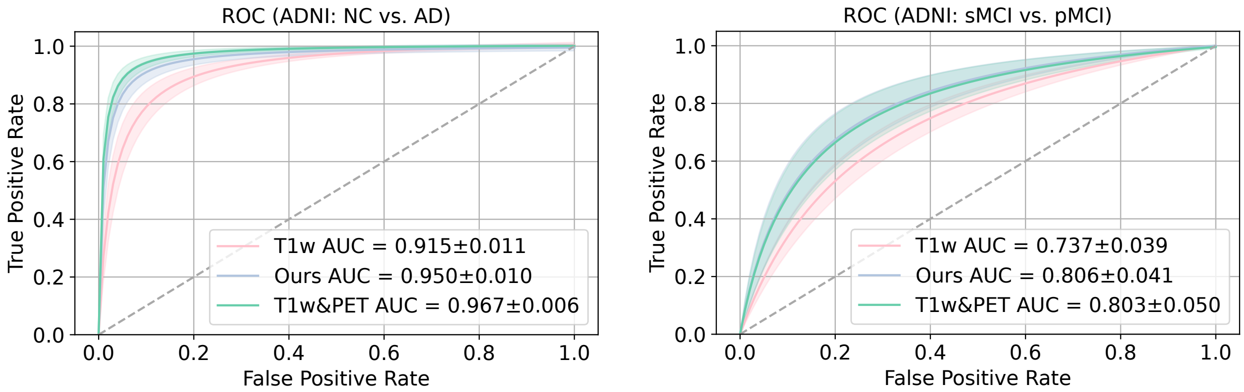

Supplementary Figure 1. Receiver operating characteristic (ROC) curves for NC *vs.* AD and sMCI *vs.* pMCI classifications based on 5-fold cross-validation on ADNI dataset. Standard deviation is depicted in shaded regions. Our framework (T1w enhanced by synthesized features of PET_{FDG} and PET_{AV45}) outperforms the single-modal 3D CNN (T1w) significantly, and shows close AUC as using real multi-modal data (T1w & PET_{FDG} & PET_{AV45}).

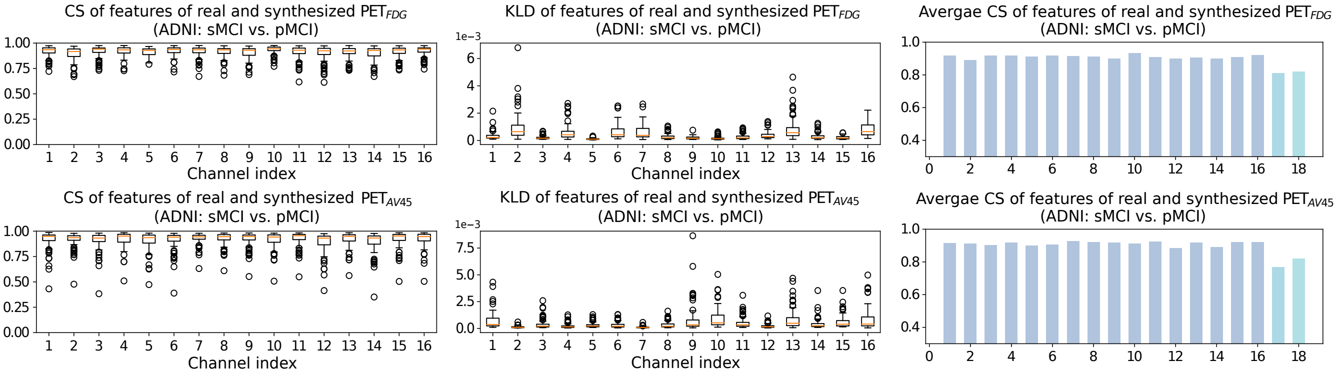

Supplementary Figure 2. Similarity measure between feature maps of real modalities, i.e., PET$_{FDG}$ and PET$_{AV45}$, and the corresponding synthesized ones on ADNI dataset. The boxplots demonstrate cosine similarity (CS) and KL divergence (KLD) of 16-channel features of the classification backbone for sMCI *vs.* pMCI classification on test data. The barplots show the average CS for the 16 channels of the classification backbone (in blue) and additional two bars (in dark green) for the features of the two fully connected layers in the classification head.

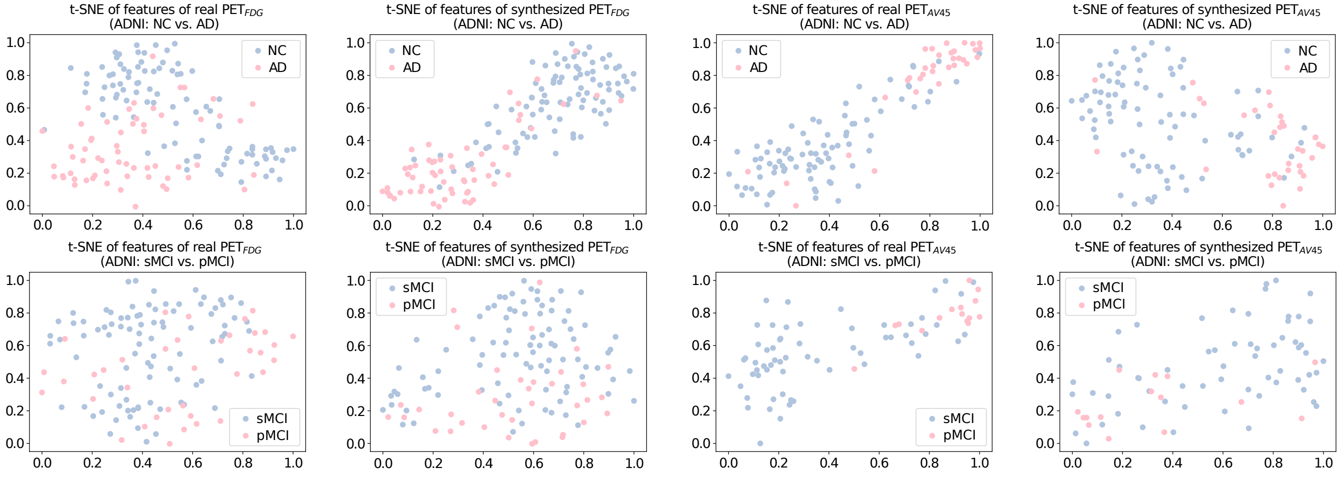

Supplementary Figure 3. T-SNE plots of feature representations of the real and synthesized modalities for different labels for NC *vs.* AD (top row) and sMCI *vs.* pMCI (bottom row) classifications.

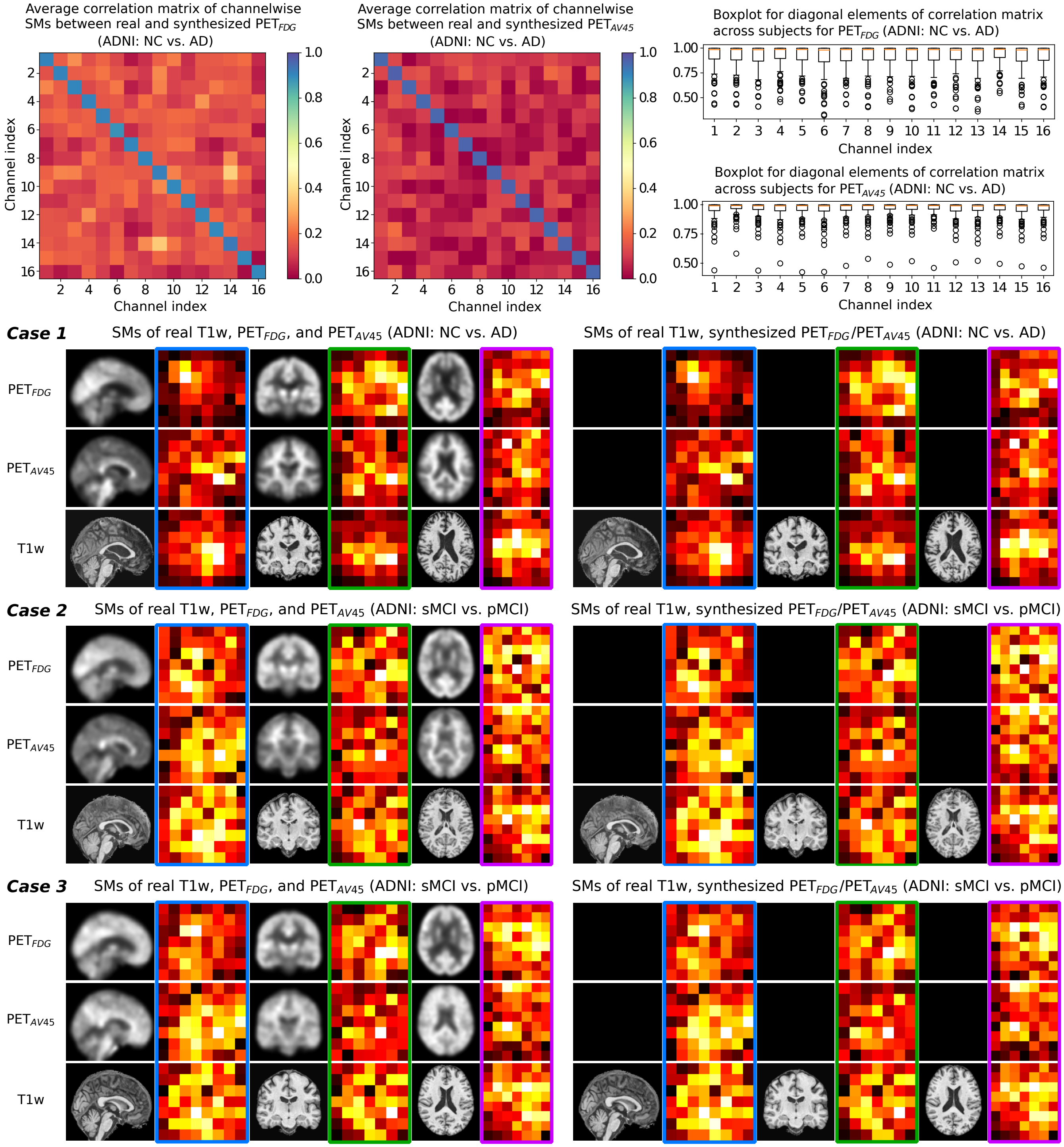

Supplementary Figure 4. Similarity measure between saliency maps (SMs) of the real and synthesized features on ADNI dataset. (a): Average correlation matrix of SMs of real and synthesized modalities for NC *vs.* AD classification. We calculate row-wise Pearson correlation coefficients between 16 SMs (for 16 backbone features) of real and synthesized modalities. We average the correlation matrices of SMs over test subjects for PET$_{FDG}$ and PET$_{AV45}$. The diagonal elements represent the correlation between real and synthesized SMs of paired channel and all the diagonal elements are close to 1, while the non-diagonal elements denote correlation between SMs of unpaired channels and are close to 0. In the right panel, we illustrate statistical analysis of the diagonal elements of the correlation matrices across test data in boxplot. Most diagonal elements are above 0.95. (b): Visualization of SMs of real and synthesized features for three cases from ADNI. The left panel illustrates SMs obtained using real multi-modal images (three views in S1), while the right panel shows those obtained via synthesized ones (in S2). The unavailable imaging modalities are masked out in black, and paired views are marked in the same color.

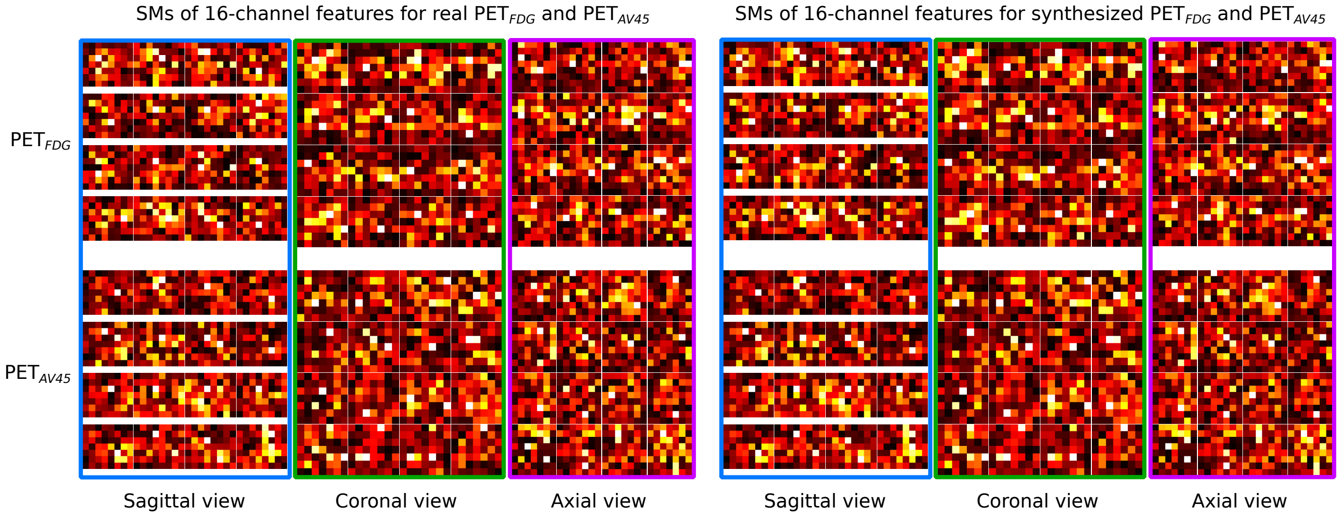

Supplementary Figure 5. The saliency maps (SMs) of the 16-channel feature maps of the real and synthesized PET$_{FDG}$ and PET$_{AV45}$ for one subject in the ADNI dataset. The left and right panels show the SMs for 16 backbone features of the real and synthesized PET, respectively, in sagittal, coronal, and axial views.

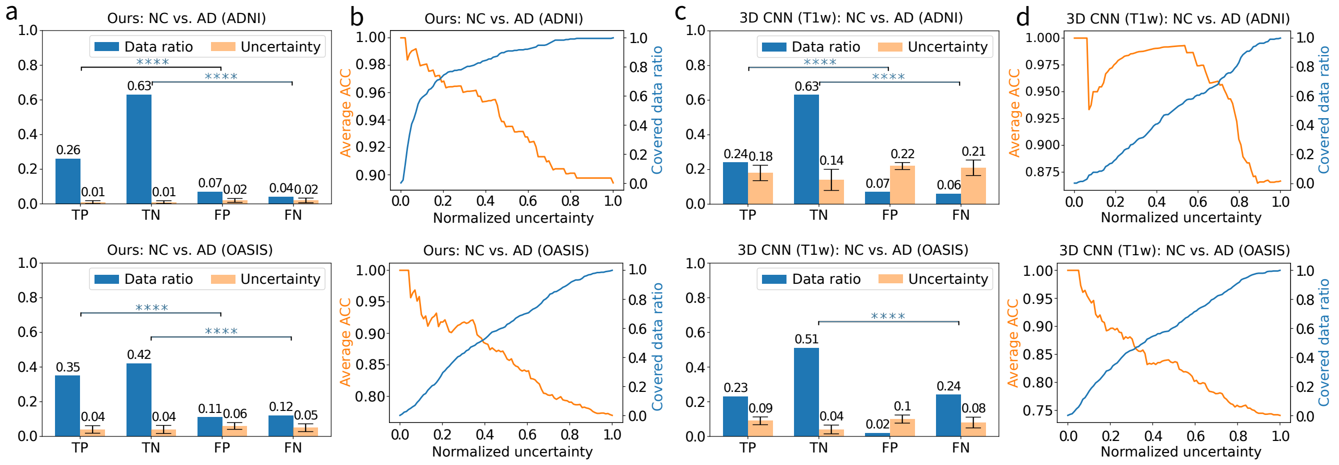

Supplementary Figure 6. Classification uncertainty analysis for NC *vs.* AD on ADNI (top row) and OASIS-3 (bottom row). (a)-(b): Uncertainty analysis for our synthesis-empowered classification framework; (c)-(d): Uncertainty analysis for the commonly used single-modal variant. (a) and (c): Plots of confusion matrix with the corresponding average uncertainty on the test data. (b) and (d): Plots of average ACC curve and covered data ratio over normalized uncertainty. We denote the two-sided p-value $p < 0.05$ as *, $p < 0.01$ as **, $p < 0.001$ as ***, and $p < 0.0001$ as ****.

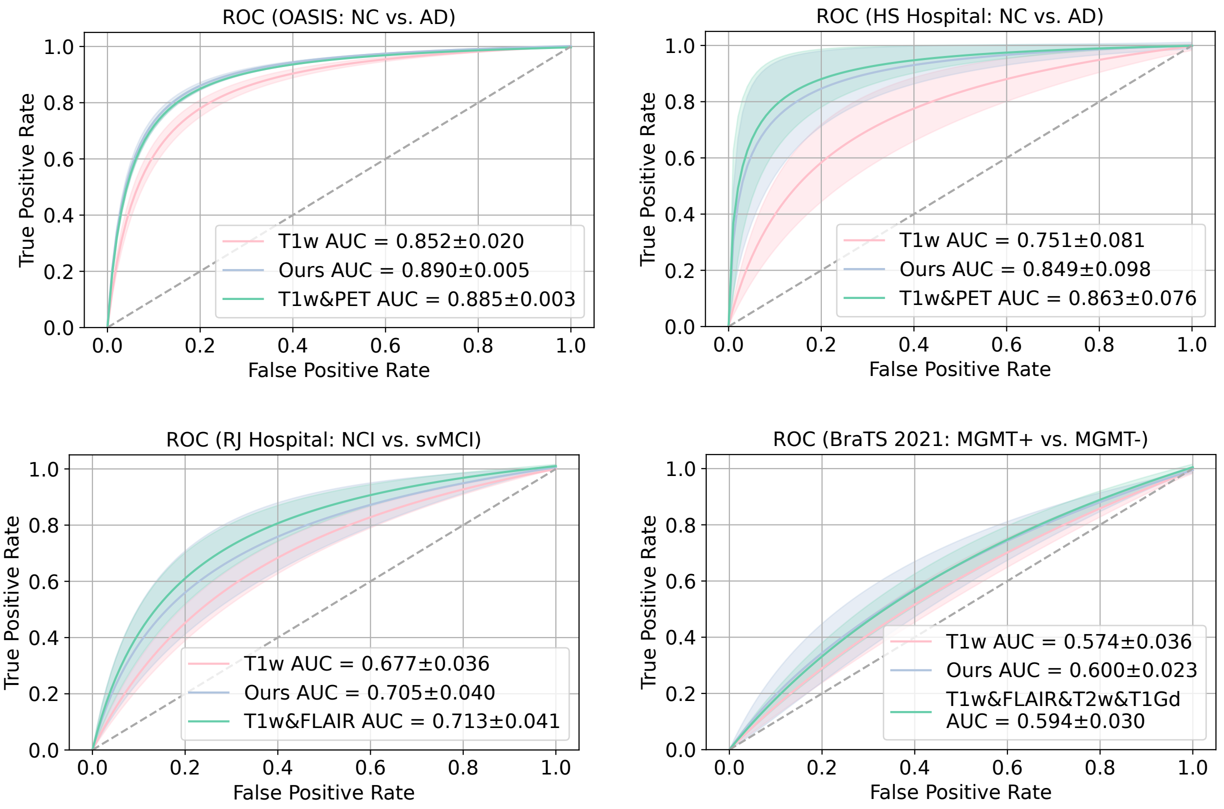

Supplementary Figure 7. Receiver operating characteristic (ROC) curves for NC *vs.* AD, NCI *vs.* svMCI, and MGMT+ *vs.* MGMT- classifications on different datasets based on 5-fold cross-validation. Standard deviation is depicted in shaded regions.
